# Bifid Median Nerve With Ulnar Division Penetrating the Flexor Digitorum Superficialis Muscle: A Case Report Discussing Two Cases

**DOI:** 10.7759/cureus.113571

**Published:** 2026-07-29

**Authors:** Ivan Chernev, Nina Chernev

**Affiliations:** 1 McLeod Orthopaedics, McLeod Regional Medical Center, Florence, USA

**Keywords:** anatomical variation, bifid median nerve, flexor digitorum superficialis muscle, neuromuscular ultrasound, wrist

## Abstract

Anatomical variations of the median nerve (MN) are very common. They may contribute to MN pathology. In addition, if not recognized, they may present a significant risk of iatrogenic nerve injury during surgical interventions. We describe two cases of an unusual bifid MN course with the ulnar division penetrating the flexor digitorum superficialis muscle. Both cases were identified during ultrasound (US) examination of the MN. US is an excellent tool that should be routinely used not only in the diagnosis of MN pathology but also to identify its anatomical variations prior to surgical procedures.

## Introduction

Anatomical variations of the median nerve (MN) course and its branches are very common [1]. Having detailed knowledge of these MN variations is critical not only for the diagnosis of MN pathology but also to avoid iatrogenic MN injury during surgical procedures. One such variation is the bifid median nerve (BMN). The BMN has been frequently discussed in the medical literature and considered a risk factor for carpal tunnel syndrome (CTS) [2]. The MN may bifurcate proximal or within the carpal tunnel. Before it bifurcates, the MN will typically descend between the flexor digitorum superficialis (FDS) and the flexor digitorum profundus (FDP) muscles. However, rare cases of an MN penetrating the FDS muscle in the forearm have been described [3,4]. The aim of this article is to report two rare cases of an unusual BMN course with only the ulnar division penetrating the FDS muscle in the distal forearm.

## Case presentation

Case 1

**Figure 1:**
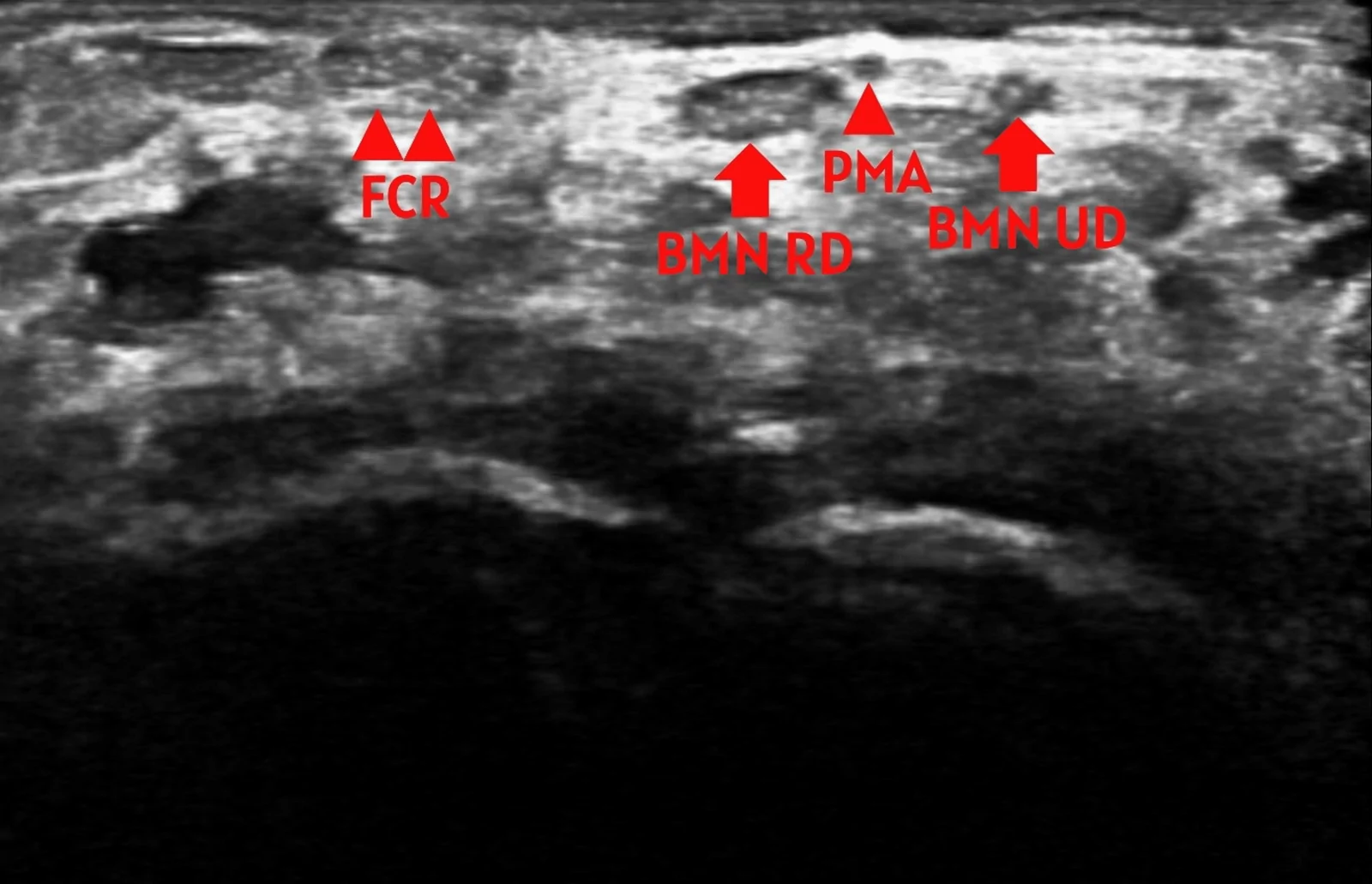
Gray scale US transverse view at the right wrist level showing BMN RD and BMN UD (arrows), PMA (arrowhead), and FCR (double arrow) US: ultrasound; BMN RD: bifid median nerve radial division; BMN UD: bifid median nerve ulnar division; PMA: persistent median artery; FCR: flexor carpi radialis

**Figure 2:**
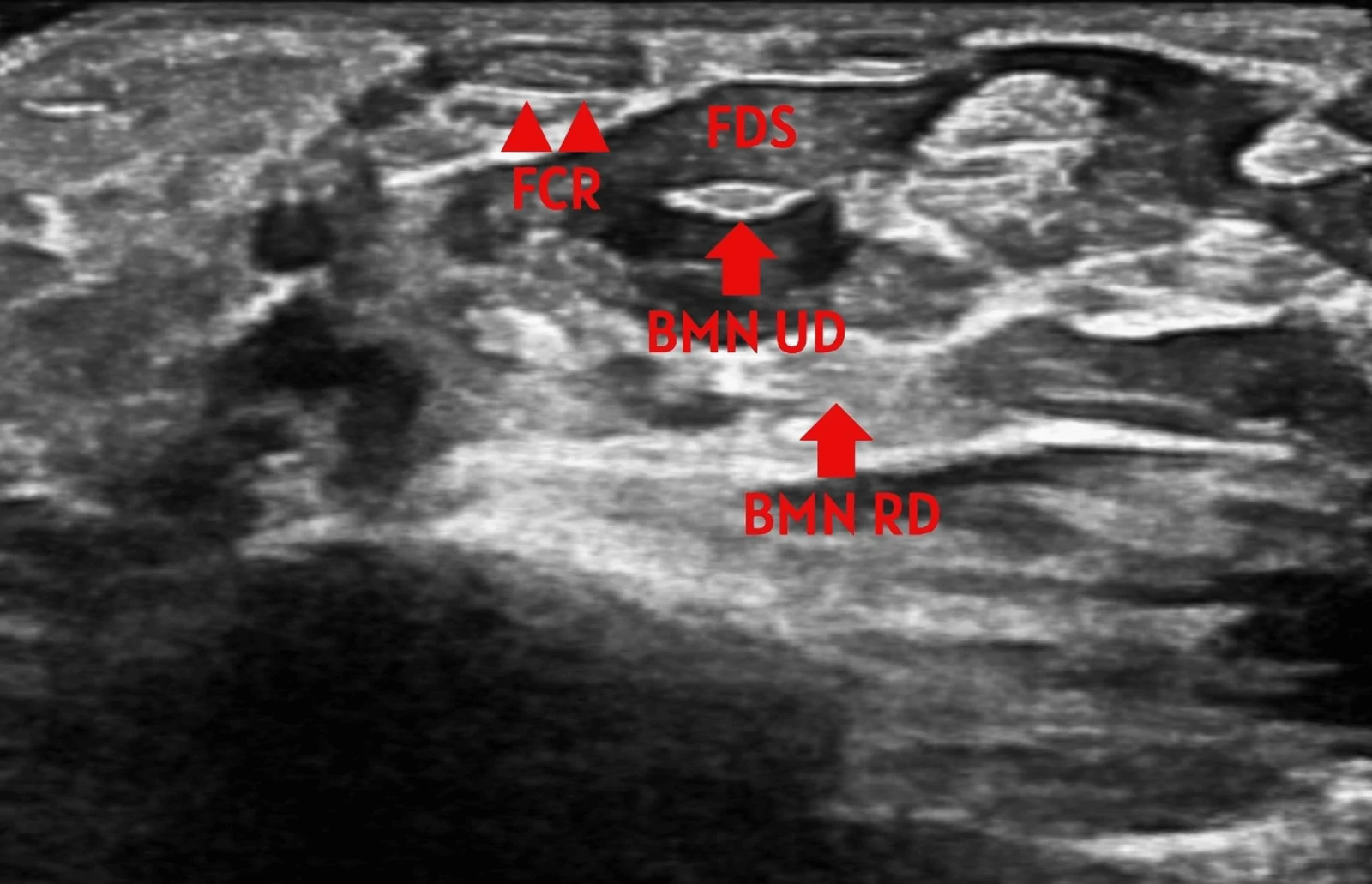
Gray scale US transverse view at the right distal volar forearm level showing BMN UD (arrow) penetrating FDS muscle, BMN RD (arrow) located dorsal to FDS muscle, and FCR (double arrow) US: ultrasound; BMN RD: bifid median nerve radial division; BMN UD: bifid median nerve ulnar division; FDS: flexor digitorum superficialis; FCR: flexor carpi radialis

A 76-year-old woman was referred to our clinic for electrodiagnostic study (EDX) with the complaint of insidious onset of bilateral hand numbness and tingling for six months. Symptoms occurred mainly at night. Symptoms during the day were minimal. Previous medical history was significant for chronic obstructive pulmonary disease, coronary artery disease, prediabetes, and Sjogren’s syndrome. Physical exam showed normal strength, sensation, and deep tendon reflexes in the upper limbs. Routine EDX showed a right mild and a left moderate median neuropathy at the wrist. Ultrasound (US) of the bilateral MN showed a normal right and a borderline increased left cross-sectional area (CSA) at the wrist with an abnormal bilateral wrist-to-forearm ratio (WFR). It also showed a right BMN and a persistent median artery (PMA) at the wrist (Figure 1). Tracking the MN proximally, the ulnar division penetrated the distal FDS muscle before joining the radial division more proximally in the distal forearm (Figure 2, Video 1).

**Video 1:** Bifid median nerve with ulnar division penetrating flexor digitorum superficialis muscle (Case 1)

Case 2

**Figure 3:**
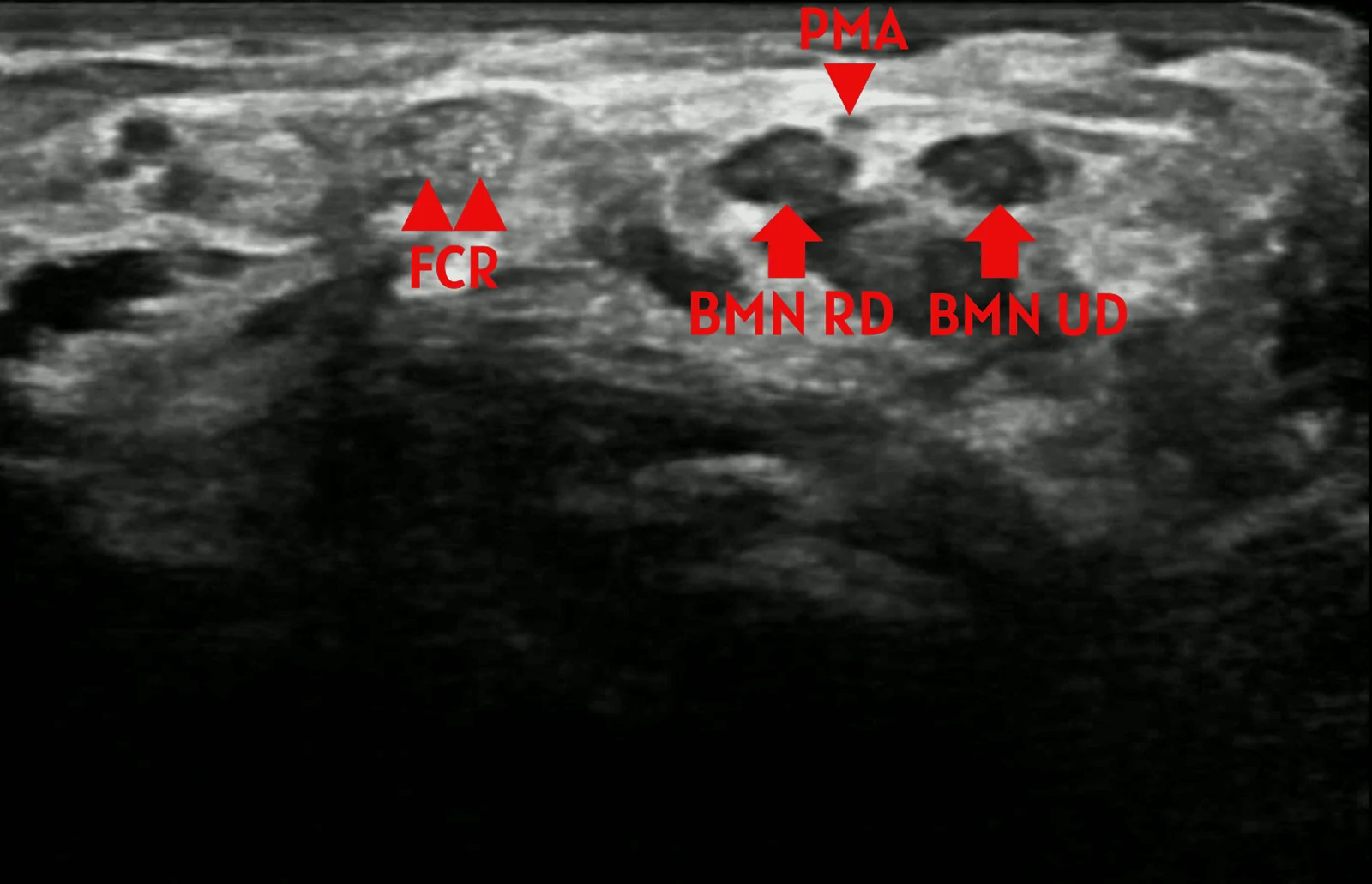
Gray scale US transverse view at the right wrist level showing BMN RD and BMN UD (arrows), PMA (arrowhead), and FCR (double arrow) US: ultrasound; BMN RD: bifid median nerve radial division; BMN UD: bifid median nerve ulnar division; PMA: persistent median artery; FCR: flexor carpi radialis

**Figure 4:**
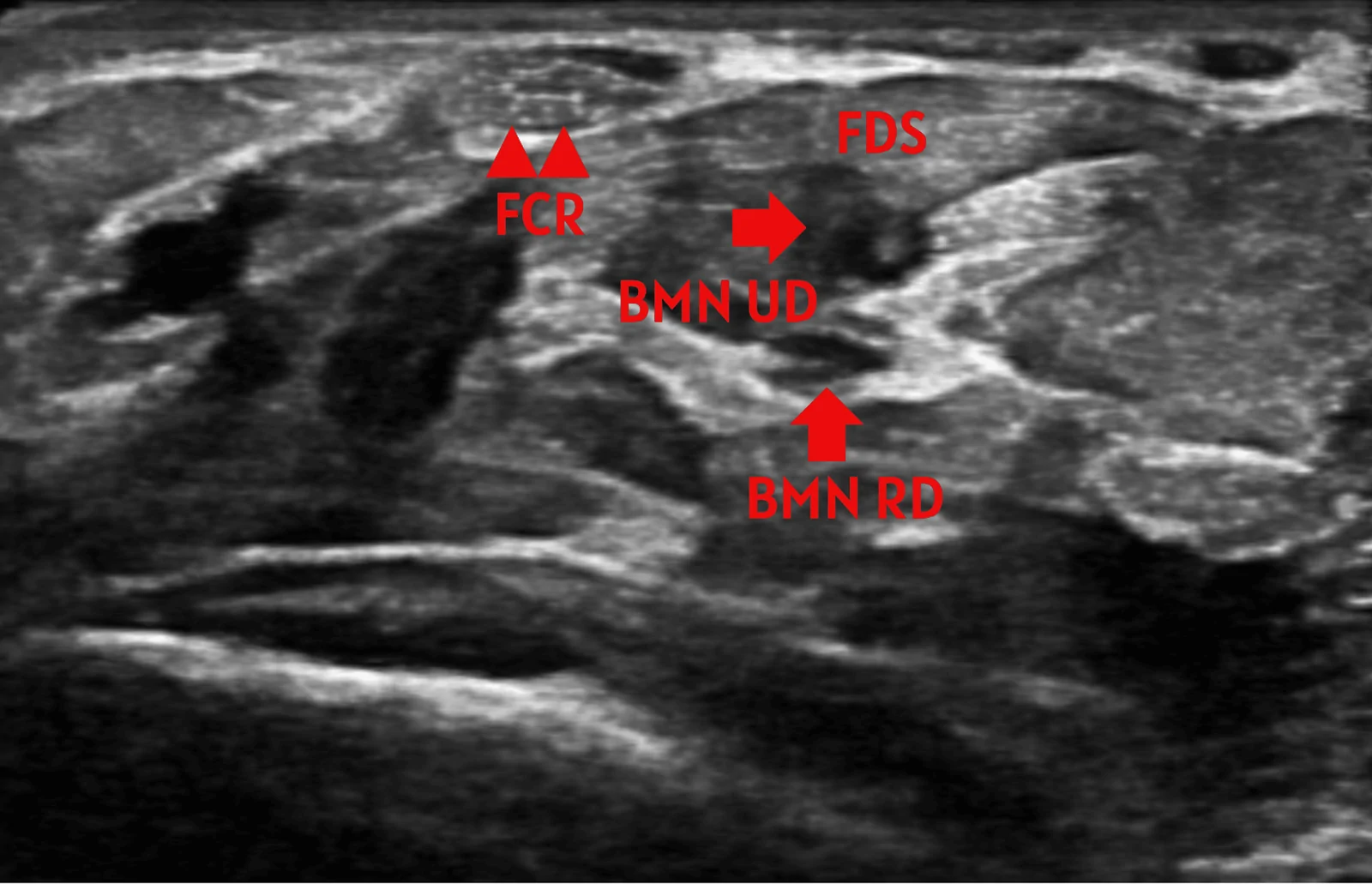
Gray scale US transverse view at the right distal volar forearm level showing BMN UD (arrow) penetrating FDS muscle, BMN RD (arrow) located dorsal to FDS muscle, and FCR (double arrow) US: ultrasound; BMN RD: bifid median nerve radial division; BMN UD: bifid median nerve ulnar division; FDS: flexor digitorum superficialis; FCR: flexor carpi radialis

A 63-year-old woman was referred to our clinic for EDX with the complaint of insidious onset of bilateral hand fourth and fifth digit intermittent numbness and tingling for several months. She also reported medial elbow discomfort. Previous medical history was significant for rheumatoid arthritis and C3-C7 cervical spine fusion. Physical exam showed normal strength, sensation, and deep tendon reflexes in the upper limbs. Routine EDX showed a left mild median neuropathy at the wrist and a right C7 chronic cervical radiculopathy. US of the bilateral ulnar nerves showed an increased bilateral CSA at the elbow. US of the bilateral MN showed a borderline increased bilateral CSA at the wrist with an abnormal bilateral WFR. It also showed a right BMN and a PMA at the wrist (Figure 3). Tracking the MN proximally, the ulnar division penetrated the distal FDS muscle before joining the radial division more proximally in the distal forearm (Figure 4, Video 2).

**Video 2:** Bifid median nerve with ulnar division penetrating flexor digitorum superficialis muscle (Case 2)

## Discussion

The MN is one of the brachial plexus terminal branches and is formed by the union of the lateral and medial cords [5]. It carries motor and sensory fibers from C6 to T1 cervical nerve roots. In the forearm, the solitary MN descends deep to the FDS muscle but superficial to the FDP muscle. It then enters the palm via the carpal tunnel, deep to the transverse carpal ligament (TCL). Normally, the MN bifurcates distally to the TCL [2]. However, it may bifurcate proximal or within the carpal tunnel, the so-called BMN [2]. BMN has been defined as partial or complete division of the MN. In partial bifurcation, the MN is subdivided by septal or fascicular separation while maintaining a shared epineural sheath. In complete bifurcation, the MN is divided into two separate components along its course [6]. BMN prevalence ranges from approximately 2.8% to 18%, depending on population characteristics and methodological approach [6]. In the most commonly used MN anatomical variations classification, Lanz categorized BMN as Group 3 [1,4,7]. However, not all anatomical MN variations and subvariations have been included in this or other published classifications, and other variations have been subsequently described in the medical literature [3,4,8]. Some of these variations describe penetration of a muscle by the MN or penetration of the MN by a muscle. Lis et al. reported a case of a solitary MN penetrating the FDS muscle in the forearm [3]. Skie and Ciocanel reported a case of the FDS muscle penetrating the MN [8]. In our cases, only the ulnar division of the BMN penetrated the distal FDS muscle. To the best of the authors’ knowledge, this variation of the MN course has been previously described in the medical literature only once [4]. Contrary to this case, an associated PMA was also present in both of our cases, increasing the anatomical complexity of the carpal tunnel. In the first case, the ulnar division was smaller than the radial division, similarly to the referenced case. In the second case, the ulnar and radial divisions were equal in size. Histologically, at the level of the wrist, the MN contains approximately 30 fascicles and nearly 22,000 nerve fibers [6]. Accordingly, different distribution patterns of fascicles may occur depending on the size of the particular MN division. Therefore, compression or injury of an individual nerve division may present with different clinical or electrophysiological findings.

Although the exact clinical significance of this particular anatomical variation is not completely clear, it may possibly predispose to MN pathology secondary to static compression, dynamic compression during muscle contraction, or possible adhesions from its intramuscular course [4]. Multiple studies have studied MN variations and their clinical significance. Jeff Walter Rajadurai et al. conducted a systematic review and meta-analysis of the clinical impact of aberrant median and ulnar nerve pathways in the forearm [9]. They concluded that these variations are frequent and clinically relevant, with a pooled prevalence of Martin-Gruber anastomosis alone of approximately 21%. They also suggested that anatomical variations in the forearm and hand can profoundly influence the results of EDX, and failure to recognize these electrophysiological patterns can result in inaccurate and misleading interpretations, potentially delaying accurate diagnosis and appropriate management. Furthermore, awareness of different anatomical variations is critical in patients with atypical clinical and electrophysiological presentation. In another systematic review and meta-analysis by Baeza et al., the authors found that MN variations in the carpal tunnel are relatively common, with a pooled prevalence of 12% [6]. They also suggested that although these variations do not appear to constitute an independent risk factor for CTS, their coexistence with vascular or muscular anomalies may influence local anatomical relationships and surgical complexity.

If nerve variations are not recognized, iatrogenic injury of the MN during surgical procedures can occur. Understanding variations of MN course and branches allows surgeons to plan incisions, dissections, and nerve reconstructions with greater precision, thereby minimizing iatrogenic injury [9]. Furthermore, release of each BMN division and excision of the intervening muscle may be required for a successful decompression and to avoid persistent entrapment neuropathy [4].

Imaging modalities such as high-resolution US and magnetic resonance imaging play a key role not only in confirming MN enlargement, shape, and altered mobility but also in identifying anatomical variations that may influence both clinical presentation and surgical planning [6]. Particularly, high-resolution US has emerged as a valuable tool for both diagnosis of MN pathology and identification of anatomical variations [6]. Studies support the complementary use of US alongside EDX testing, particularly in patients with uncharacteristic clinical and electrophysiological presentation [6].

In summary, understanding the course of the MN and its variations can improve MN pathology diagnosis and prevent surgical complications. Furthermore, there is a need for an updated classification of MN variations, and this variation should be added to the MN variations list.

## Conclusions

Anatomical variations of the MN are very common. If not recognized, they may contribute to MN pathology misdiagnosis or present a significant risk of iatrogenic nerve injury during surgical interventions. Clinicians across different medical specialties should suspect MN variations in patients with atypical clinical presentation and EDX findings. Complementary use of US alongside EDX testing is an excellent tool not only to diagnose MN pathology, such as median neuropathy at the wrist or more proximally, but also to identify MN anatomical variations in preoperative surgical planning.

## References

[1] Theofilopoulou S, Katouni K, Papadopoulos V (2023). Variations of the median nerve and carpal tunnel syndrome: a systematic review of the literature. Maedica (Bucur).

[2] Asghar A, Patra A, Satish Ravi K, Tubbs RS, Kumar A, Naaz S (2022). Bifid median nerve as an anatomical risk factor for carpal tunnel syndrome: a meta-analysis. Clin Anat.

[3] Lis M, Solewski B, Koziej M (2018). The median nerve penetrating through the radial head of the flexor digitorum superficialis muscle: case report. Folia Med Cracov.

[4] Crist J, Glazebrook K, McKenzie G (2020). Incidental bifid median nerve with a wandering ulnar division separated by the third flexor digitorum superficialis muscle: importance of ultrasound evaluation. Radiol Case Rep.

[5] (2026). Anatomy, shoulder and upper limb, median nerve. https://www.statpearls.com/point-of-care/24837.

[6] Baeza PN, Navia-Ramirez MF, Lobos-Ortiz M (2026). Prevalence and clinical implications of median nerve variants within the carpal tunnel: a systematic review and meta-analysis. Curr Probl Surg.

[7] Lanz U (1977). Anatomical variations of the median nerve in the carpal tunnel. J Hand Surg Am.

[8] Skie M, Ciocanel D (2010). Anomaly of flexor digitorum superficialis penetrating through the median nerve: case report. J Hand Surg Am.

[9] Jeff Walter Rajadurai OR, Prem Regis PA, Supadevi S, Vinoth S (2026). Dissecting neural anomalies and clinical impact of aberrant median and ulnar nerve pathways in the forearm: a systematic review and meta analysis. J Hand Microsurg.

